# Classification of Alzheimer’s Disease Using Maximal Information Coefficient-Based Functional Connectivity with an Extreme Learning Machine

**DOI:** 10.3390/brainsci13071046

**Published:** 2023-07-08

**Authors:** Nishant Chauhan, Byung-Jae Choi

**Affiliations:** Department of Electronic Engineering, Daegu University, Gyeongsan 38453, Republic of Korea; nishantsep1090@daegu.ac.kr

**Keywords:** functional connectivity, fMRI, alzheimer’s disease, pearson correlation, maximal information coefficient, machine learning, deep learning, extreme learning machine

## Abstract

Alzheimer’s disease (AD) is a progressive chronic illness that leads to cognitive decline and dementia. Neuroimaging technologies, such as functional magnetic resonance imaging (fMRI), and deep learning approaches offer promising avenues for AD classification. In this study, we investigate the use of fMRI-based functional connectivity (FC) measures, including the Pearson correlation coefficient (PCC), maximal information coefficient (MIC), and extended maximal information coefficient (eMIC), combined with extreme learning machines (ELM) for AD classification. Our findings demonstrate that employing non-linear techniques, such as MIC and eMIC, as features for classification yields accurate results. Specifically, eMIC-based features achieve a high accuracy of 94% for classifying cognitively normal (CN) and mild cognitive impairment (MCI) individuals, outperforming PCC (81%) and MIC (85%). For MCI and AD classification, MIC achieves higher accuracy (81%) compared to PCC (58%) and eMIC (78%). In CN and AD classification, eMIC exhibits the best accuracy of 95% compared to MIC (90%) and PCC (87%). These results underscore the effectiveness of fMRI-based features derived from non-linear techniques in accurately differentiating AD and MCI individuals from CN individuals, emphasizing the potential of neuroimaging and machine learning methods for improving AD diagnosis and classification.

## 1. Introduction

One of the most commonly reported causes of dementia in the elderly is AD, which is a chronic, irreversible neurological disease [1]. It is a degenerative, inevitable, progressive neurological disorder and complex disease that continually damages brain cells, leading to memory and cognitive skills loss and, eventually, the inability to carry out the most basic activities. This condition causes cognitive deterioration, which eventually leads to dementia. In the context of neurodegenerative dementia, such as AD, the initial stages are characterized by mild deterioration, which progressively worsens over time. The diagnosis of AD cannot be made solely through a single test. Instead, healthcare professionals gather comprehensive information on a patient’s medical and mental health history, as well as their family background. Additionally, they engage in consultations with relatives and conduct neurological and cognitive tests. To exclude other potential causes of dementia, participants undergo additional assessments, including blood tests and brain imaging. The process of data collection and physician interpretation can take several weeks [2]. In the AD development stage, MCI represents a small cognitive decline in mental skills, which shows the pathophysiology from cognitively normal to AD. Eventually, more than 33 percent of subjects with MCI will develop AD within five years or more [3,4]. Early diagnosis of individuals with MCI before the emergence of AD is crucial for the effectiveness of potential treatments. This is because patients in the MCI stage do not exhibit the same level of significant brain damage as those already diagnosed with AD. Determining a comprehensive treatment plan for AD poses challenges due to its unknown cause and mechanisms. However, a combination of medication, exercise, and memory training has shown potential in slowing the progression of the disease. Precise diagnosis of MCI and early detection of AD are crucial in order to delay disease advancement and enhance the patient’s overall quality of life [5].

Neuroimaging techniques are commonly employed in research due to their high precision and accuracy in diagnosing AD. For example, statistical models and MRI are frequently utilized to predict early stages of AD [6]. While neuroimaging methods offer powerful diagnostic capabilities, their effective analysis requires specialized expertise. Therefore, it is crucial to enhance neuroimaging techniques to further support the diagnosis of AD. The fMRI technique is a non-invasive approach that utilizes blood oxygen level-dependent (BOLD) signals to assess hemodynamic changes induced by neuronal activity. The fMRI technique in neuroimaging employs cerebral blood flow measurements to determine how the brain is functioning. It is primarily utilized to visualize the activation patterns and temporal interactions among different brain regions. It is believed that functional changes in the brain occur before the appearance of anatomical abnormalities [7]. Resting-state FC measurements, which involve fMRI scans without specific tasks or stimuli, have been previously shown to be effective in detecting and predicting the early stages of AD [8].

Here are the key points explaining how imaging techniques provide significant advantages in AD diagnosis:

1. Enhanced visualization: Imaging techniques provide clear and detailed visualization of structural and functional brain abnormalities associated with AD.
2. Non-invasiveness: Imaging techniques offer a non-invasive approach, ensuring patient comfort and allowing for repeated examinations.
3. Early detection: Imaging can identify subtle brain changes even before the onset of clinical symptoms, enabling early detection and intervention.
4. Comprehensive assessment: Imaging captures both structural and functional aspects of the brain, providing a comprehensive evaluation of key pathological hallmarks and assessing connectivity and activity patterns related to cognitive decline.
5. Personalized medicine: By examining individual brain characteristics, imaging allows for tailored diagnostic and treatment approaches, considering unique variations in disease presentation.

By analyzing alterations in the composition of deoxygenated hemoglobin in the regional blood supply, neural activity patterns in specific brain regions can be assessed [9].

FC refers to the temporal correlation between neurophysiological variables measured in different brain regions, serving as a measure of the interactions between these regions [10,11]. Among the various analytical methods for resting-state fMRI, FC analysis is widely utilized. This method statistically demonstrates the synchrony of functional activation between non-adjacent brain regions, providing insights into their functional relationships. FC has definitively demonstrated its significance in investigating functional interconnections across different brain areas. Due to the use of linear FC in examining the brain’s operating processes, we now have a better insight into how distinct brain areas interact [12]. Hence, resting-state fMRI-computed FC can be used to investigate the possibility that FC can serve as a predictor in AD patients, including those with CN or MCI.

Numerous studies have investigated FC between brain regions using the PCC, which provides a linear estimation of the relationship between two random elements [13]. However, linear correlation analyses alone may not sufficiently capture the intricate connections between brain regions. Therefore, in the context of AD patients, utilizing fMRI and non-linear FC measures may be more suitable for capturing these intricate connections [14].

One potential non-linear measure is MIC, which has been recognized as a valuable tool for assessing the relationship between two time variables [15] and as an effective approach for reconstructing the functional network of the brain [16,17]. To further explore non-linear relationships, the extended MIC (eMIC) combines MIC with PCC, allowing for the assessment of non-linear connections between two elements [18].

Artificial intelligence (AI) and machine learning (ML) offer significant advancements in the diagnosis of brain diseases such as AD. By leveraging AI algorithms and ML models, medical professionals can analyze diverse data sources, including imaging scans, genetic profiles, and clinical records, to uncover complex patterns and biomarkers associated with AD. These technologies enhance early detection and enable accurate prediction of disease progression, aiding in the development of personalized treatment plans. Additionally, AI and ML provide valuable decision support tools, empowering healthcare providers with evidence-based insights for more precise and efficient diagnoses. Deep learning (DL) algorithms differ from traditional ML approaches. While ML models continue to improve incrementally, they still require human intervention and adjustment when inaccurate predictions are made. In contrast, DL models leverage their neural networks to autonomously evaluate prediction accuracy. This capability enables DL algorithms to effectively handle unstructured data and reduces the need for extensive feature engineering, which is essential for ML models. DL algorithms have the capability to estimate an optimal data representation of raw images, eliminating the need for extensive feature engineering and enabling a more independent and object-oriented approach. This advantage stems from their minimal image preprocessing requirements. Consequently, DL algorithms have demonstrated enhanced effectiveness in detecting both fine and extensive anatomical abnormalities. Furthermore, DL algorithms have achieved optimal performances across various domains, including natural language understanding, computer vision, and speech recognition tasks, as well as more recent applications in MRI analysis [19], X-rays [20], CT scans [21], PET [22], and EEG [23].

In contrast, traditional ML techniques typically follow specific pipelines or steps for image analysis. These steps include data preprocessing, data augmentation, segmentation, feature extraction, and classification. Furthermore, ML algorithms often rely on a substantial amount of high-quality data to achieve highly accurate results. Therefore, it is strongly recommended to prepare the data appropriately and, if necessary, take additional steps to ensure optimal results. In 2012, researchers introduced an advanced convolutional neural network (CNN) known as AlexNet [24]. This study utilized complete brain fMRI scans as part of their research. Subsequently, other researchers employed MRI data and pre-trained CNN models, including 3D convolutional autoencoders, for binary and multiclass classification tasks such as AD/MCI vs. NC (normal control), AD vs. MCI, MCI vs. NC, and AD vs. MCI vs. AD. Additionally, they utilized the LeNet model to differentiate AD from NC [25]. Several subsequent studies continued to leverage the CNN approach, incorporating a combination of MRI and PET brain scans [26,27]. In another study [28], the authors introduced a novel strategy by implementing a deep belief network (DBN) that accepts 3D patches as input. They further employed a support vector machine (SVM) to classify gray and white matter areas extracted from MRI and PET scans, with the objective of distinguishing between NC and AD.

Motivated by the previously mentioned characteristics and research findings, our study aims to contribute to the field of AD research. Specifically, we intend to analyze fMRI data using the PCC, MIC, and eMIC to evaluate both linear and non-linear FC measures. Our objective is to investigate the discriminative abilities of linear and non-linear FC for different cognitive levels among CN, MCI, and AD patients based on their specific characteristics.

To accomplish this, we constructed correlation matrices based on the FC values between distinct regions of interest (ROIs), effectively representing the brain network. These matrices depict the relationships between different brain regions, forming a graph-like structure. Notably, this graph exhibits non-Euclidean properties. To address these structural invariances, we utilized graph embedding techniques, employing node2vec, which transforms graph data into vectors or sets of vectors.

Finally, we utilized the MLELM classifier to distinguish AD participants from individuals with CN and MCI. This classification was achieved by leveraging the extracted graph-based features obtained through the previously mentioned FC analyses and graph embedding approach.

## 2. The Pathophysiology of Alzheimer’s Disease

The most reliable diagnostic method for AD is currently based on pathological examination. Although several macroscopic characteristics of AD can be identified, no single characteristic or combination of characteristics can definitively diagnose the disease. However, certain features strongly indicate the presence of AD.

**Figure 1:**
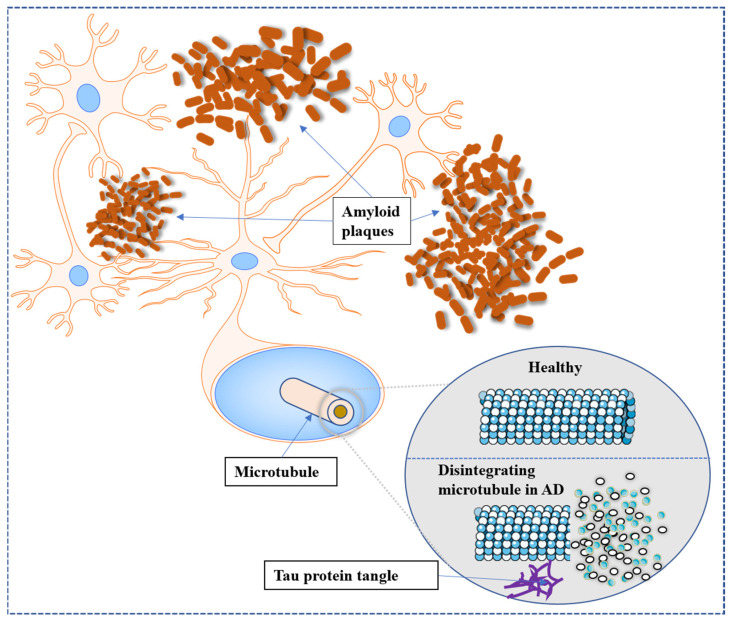
Pathophysiology of AD in the brain. The metabolism of APP sometimes follows a non-amyloidogenic pathway and forms amyloid plaques. Tau, a microtubule-associated protein, generates insoluble filaments that congregate as neurofibrillary tangles in AD.

In the human body and brain, the protein amyloid naturally develops. In AD, normal amyloid groups undergo structural changes that disrupt their normal functioning. Abnormal amyloid groups can trigger alterations in nearby healthy amyloid groups, resulting in the formation of large clusters called plaques. These plaques, depicted as the brown, cloud-like substance in Figure 1, are associated with the formation of brain lesions, which are a distinctive feature of AD and contribute to the degeneration of brain cells [28,29]. The hippocampus, a brain region particularly susceptible to plaque formation, plays a crucial role in the processing of short-term to long-term memories. Damage to the hippocampus has been linked to the symptoms of AD. Another protein naturally present in the human body and brain is tau. Its primary function in the brain is to maintain the stability of brain cell axons, which are tube-like structures through which electrical impulses pass. In AD, tau proteins undergo structural changes that lead to their aggregation with other tau fibers. This entanglement of tau proteins disrupts the stability of brain cell axons, resulting in their degeneration and eventual death [30]. Hyperphosphorylation of tau disrupts its normal function in stabilizing microtubules, leading to the formation of neurofibrillary tangles. These tangles contribute to neuronal dysfunction and ultimately result in cognitive decline and neurodegenerative diseases, such as Alzheimer’s disease. Understanding the role of tau hyperphosphorylation in disease progression is crucial for developing targeted therapies and interventions to mitigate its detrimental effects. The tau tangles, depicted in purple in Figure 1, interfere with the transmission of signals between neurons, thereby disrupting synaptic communication.

Numerous studies have provided evidence that microwave radiation poses risks to the human brain [31]. The exposure to microwaves can induce protein damage and disrupt mitochondrial activity by affecting the generation of reactive oxygen species (ROS) and levels of adenosine triphosphate (ATP). These effects can result in DNA damage, including breaks in single- and double-stranded DNA, and contribute to the development of neurodegenerative diseases, including AD [32].

## 3. Materials and Methods

**Figure 2:**
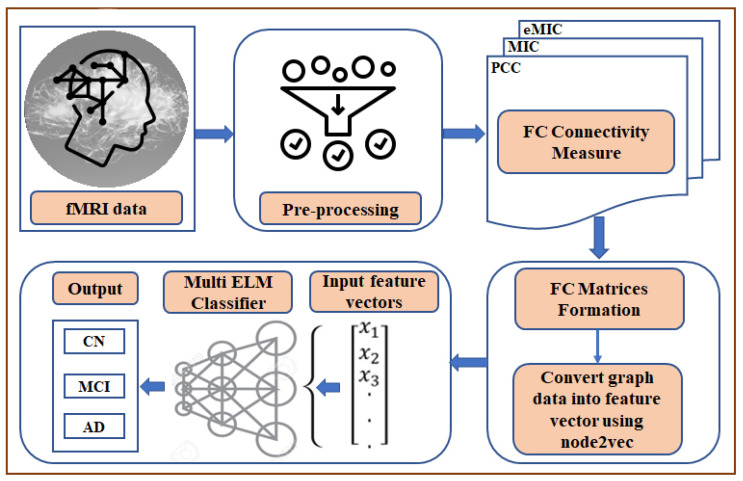
Architecture of proposed method.

The proposed method, as depicted in Figure 2, consists of several steps to classify AD versus CN, CN versus MCI, and AD versus MCI. The first step involves retrieving the fMRI data from the Alzheimer’s Disease Neuroimaging Initiative (ADNI) database. The fMRI data are then subjected to standard preprocessing steps, including motion correction, slice timing correction, normalization, and spatial smoothing, to ensure data quality and consistency.

Next, FC measures are computed using the PCC, as well as the MIC and eMIC correlation methods. These measures capture the statistical dependencies between brain regions and provide insights into the underlying functional networks. The FC matrices are formed based on these measures, representing the connectivity patterns between different regions of the brain.

To facilitate further analysis, the FC measure graph data are transformed into vector data using the node2vec method. Node2vec leverages the concept of random walks in the graph to generate embedding vectors that capture the structural properties of each node in the graph. These vectors serve as the input to the M-ELM for classification tasks.

### 3.1. Dataset

One of the most widely used datasets for diagnosing AD is the ADNI dataset [33]. ADNI is a long-term study that aims to investigate the use of serial MRI, CSF measures, PET, clinical assessments, and other neuropsychological criteria for tracking MCI and early AD progression. The identification of sensitive and accurate markers for early AD diagnosis in this study will benefit clinical specialists and researchers in developing effective treatments, monitoring treatment efficacy, and reducing the time and cost of clinical tests. The principal investigator of the initiative is Michael W. Weiner, MD, from the University of California and VA Medical Center. ADNI involves collaboration between numerous co-investigators from various academic institutions and corporate enterprises, with subjects recruited from over 50 sites across the United States and Canada.

In this study, we utilized the ADNI dataset, which includes 1534 patients and 402,446 resting-state functional MRIs (rs-fMRI) [33]. The dataset used in our analysis consisted of a total of 383 patients (male: 223, female: 160), including 135 patients with CN, 148 patients with MCI, and 100 patients with AD labels. The average age of the patients was 75.8. After the image extraction process, a total of 4364 MR images were acquired, and 58 unnecessary images were removed through data cleaning.

For preprocessing the original fMRI data, we employed Statistical Parametric Mapping software (SPM8) in Matlab [34]. The preprocessing procedures involved slice timing, normalization, realignment to the Montreal Neurological Institute (MNI) space, and smoothing with a Gaussian kernel. Subsequently, the REST toolkit, an open source software (https://www.nitrc.org/projects/rest/, accessed on 1 July 2023) was used to filter the data within a low-frequency range of 0.01–0.08 Hz to eliminate very low-frequency drift and high-frequency noise [35]. Table 1 provides a description of the dataset after applying image preprocessing techniques.

### 3.2. FC Matrix Formation

The preprocessed fMRI scan data were parcellated into 116 brain areas using the Automated Anatomical Labeling (AAL) template from the WFU Pick Atlas program [36]. Subsequently, three FC matrices were generated for each subject by computing individual brain FC using PCC, MIC, and eMIC. Each FC value between two brain regions was considered a feature. 

The PCC between two variables is calculated using the covariance and the product of the standard deviations of the two variables, which can be expressed as [13]

(1) $$\rho_{AB}=\frac{cov(A,B)}{\sigma_{A}\sigma_{B}}=\frac{E\left[\left(A-\mu_{A}\right)\left(B-\mu_{B}\right)\right]}{\sigma_{A}\sigma_{B}}$$

Here, cov⁡(A,B) is the covariance between two variables *A* and *B*. $\sigma_{A}$ and $\sigma_{B}$ are the standard deviation of *A* and *B*, respectively. $\mu_{A}$ and $\mu_{B}$ correspond to the means of *A* and *B*, respectively. 

The mutual information of two random variables is defined as follows:

(2) $$\begin{array}{c} I(A,B)=H(A)+H(B)-H(A,B)\\ =H(A)-H(A|B)\\ =H(B)-H(B|A) \end{array}$$

where *H*(*A*) and *H*(*B*) are the marginal entropies of *A* and *B*, respectively. *H*(*A*|*B*) and *H*(*B*|*A*) correspond to the conditional entropies, and *H*(*A*, *B*) is the joint entropy of *A* and *B*. Thus, the $m_{A\times B}$ of the characteristic matrix can be expressed as

(3) $$m_{A\times B}=\frac{max\left\{I(A,B)\right\}}{logmin\{(n_{A},n_{B})\}}$$

Here, I(A,B) is the MI of the probability distribution function. *n* is the number of data points, and $n_{A}$ and $n_{B}$ are the number of bins of the partition. MIC can be calculated as the maximum $m_{A\times B}$ over all ordered pairs (*A*, *B*). Thus, MIC can be expressed as [14]

(4) $$MIC=\underset{AB<n}{max}\left\{m_{A\times B}\right\}$$

eMIC is an estimate of the non-linear correlation between two variables *A* and *B* that can be expressed as [18]

(5) $$\mathrm{eMIC}=\mathrm{MIC}-\rho^{2}$$

where ρ corresponds to the PCC of the two variables.

The technique of network embedding serves as a dimensionality reduction tool that transforms networks into vector spaces. In our study, we focus specifically on nodal embedding, which involves mapping a graph into a set of vectors, where each vector corresponds to a specific vertex in the graph. To achieve the vector representation of the graph, we employ the node2vec method, which has demonstrated its ability to capture the structural similarities of nodes [37]. This method utilizes the skip-gram architecture, which learns to generate feature representations of words based on their surrounding context. In the context of networks, the notion of context is translated into the concept of neighborhood. Node2vec precisely captures the flexible notion of a node’s neighborhood, considering various properties of interest, such as structural or relational similarities between neighborhoods.

### 3.3. Classification

**Figure 3:**
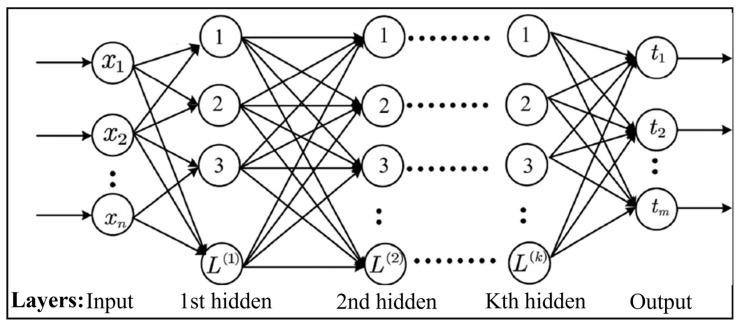
The architecture of multiple hidden layer extreme learning machine.

The extreme learning machine (ELM) is a single hidden layer feedforward neural network (SLFN) that demonstrates faster convergence rates compared to traditional approaches, resulting in remarkable outcomes [38,39]. The SLFN randomly selects hidden layer weights, and the Moore–Penrose inverse is used to analytically estimate the parameters of the output layer [40]. As a result, the tuning of hidden layer parameters does not require gradient-based backpropagation. This enables exceptionally fast training, making it particularly suitable for analyzing big data. ELM offers several advantages over conventional neural networks and support vector machines, including rapid learning, straightforward implementation, and minimal user intervention [41]. However, due to its shallow architecture, feature learning with ELM approaches may not be practical for certain applications, even with a large number of hidden nodes. In this study, we utilized a multilayer ELM as described in [42]. As illustrated in Figure 3, each layer is connected to the layer above it in a feedforward manner.

The multilayer ELM architecture, as depicted in Figure 3, establishes a feedforward connection between each layer and the one above it. By introducing additional layers, the multilayer ELM expands the depth of the network, enabling enhanced feature learning capabilities. The Algorithm 1 of the multi-layer ELM can be outlined as follows [42]:
**Algorithm 1** Multi-layer ELM Algorithm**Input:** Training data (X) with corresponding labels (Y), number of hidden layers (L), and the number of neurons in each hidden layer ($N_{l}$).
Initialize the input-to-hidden layer weights randomly for each layer l from 1 to L.For each layer l from 1 to L, compute the hidden layer output $H_{l}$ using the following equation:
$$H_{l}=g_{l}\left(X\times W_{l}\right)$$
where $g_{l}$ is the activation function of layer l, X represents the input data, and $W_{l}$ is the weight matrix of layer l.Concatenate the outputs of all hidden layers to obtain the final hidden layer output H.Compute the output weights β using the equation:
β=pinv(H) x Y
where pinv(H) is the Moore–Penrose pseudoinverse of the hidden layer output H.
**Output:** The trained M-ELM model with the calculated output weights β.

The MLELM algorithm allows for efficient training of deep architectures, benefiting from the fast learning properties of ELM while leveraging the representational power of multiple hidden layers. This enables MLELM to effectively capture intricate patterns and extract high-level features from complex datasets, enhancing its classification performance.

The performance of the classifier was evaluated using three performance metrics: accuracy, sensitivity, and specificity. The percentage of participants that were correctly classified is measured by accuracy, while sensitivity and specificity are used to evaluate the true positive (TP) and true negative (TN) rates. These two parts represent the rightfully identified subjects. Both false positives (FP) and false negatives (FN) suggest subjects that were misclassified. 

Accuracy is measured by computing the ratio of a classifier’s correctly classified examples using equation [43]:

(6) $$\mathrm{Accuracy}\;=\frac{TP+TN}{TP+TN+FP+FN}$$

This result may not serve as an absolute performance metric when the class distribution of the dataset is unstable. For instance, if class C1 significantly outweighs class C2, a classifier that labels all examples as belonging to class C1 could yield a high accuracy value. Sensitivity refers to the rate of true positives (TP), while specificity denotes the rate of true negatives (TN). Sensitivity and specificity can be defined as follows:

(7) $$\mathrm{Sensitivity}=\frac{TP}{TP+TN+FP+FN}$$

(8) $$\mathrm{Specificity}=\frac{TN}{TP+TN+FP+FN}$$

Sensitivity is concerned with the proportion of correctly identified patients, also known as the true positive rate. It measures the ability of a classifier to correctly identify positive instances from the total actual positive instances. On the other hand, specificity relates to the proportion of correctly identified controls, also referred to as the true negative rate. It measures the ability of a classifier to correctly identify negative instances from the total actual negative instances. Sensitivity and specificity are crucial performance measures in classification tasks, as they provide insights into how well a classifier can accurately distinguish between different classes.

The effectiveness of classifiers and feature selection approaches was assessed overall using a 10-fold cross-validation method. First, we divided the subjects into ten equal groups (folds), with 10% of the test subjects and 90% of the training subjects in each fold. The top-ranked features retrieved by PCC, MIC, and eMIC were used to train the classifier in the form of FC matrices, which were then converted into feature vectors. We calculated the average cross-validated accuracy as well as sensitivity and specificity.

## 4. Results and Discussion

In this paper, the PCC, MIC, and eMIC methods were utilized to identify significantly diverse linear and non-linear FC using resting-state fMRI data from AD patients. Patients with AD were divided into three groups based on their cognitive levels: CN, MCI, and AD. 

**Figure 4:**
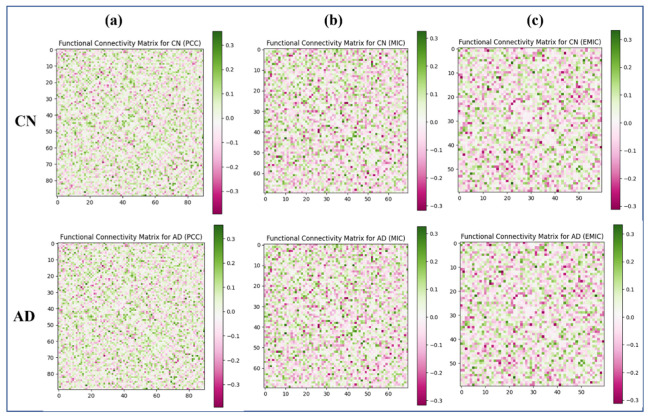
Functional connectivity matrices based on PCC, MIC, and eMIC (for CN and AD group). (**a**) FC Matrices of CN and AD group using PCC (**b**) FC Matrices of CN and AD group using MIC (**c**) FC Matrices of CN and AD group using eMIC.

Each participant’s FC matrix was generated using PCC, MIC, and eMIC, measuring the statistical degree of connectivity across brain regions (Figure 4). 

Using PCC, MIC, and eMIC, we evaluated the total brain resting-state FC matrices of AD patients and CN. The FC matrices of the CN (Figure 3, the first row) and the AD patients (Figure 3, the second row) derived using the PCC, MIC, and eMIC methods are shown. The FC matrices generated using the PCC, MIC, and eMIC methods were transformed to their vector representation using the node2vec method, and these data are further used for classification using multilayer ELM. The number of hidden layer nodes utilized has a significant impact on the multilayer ELM classifier’s performance. We used 1000 hidden layers to generate highly accurate performance results in this experiment.

**Figure 5:**
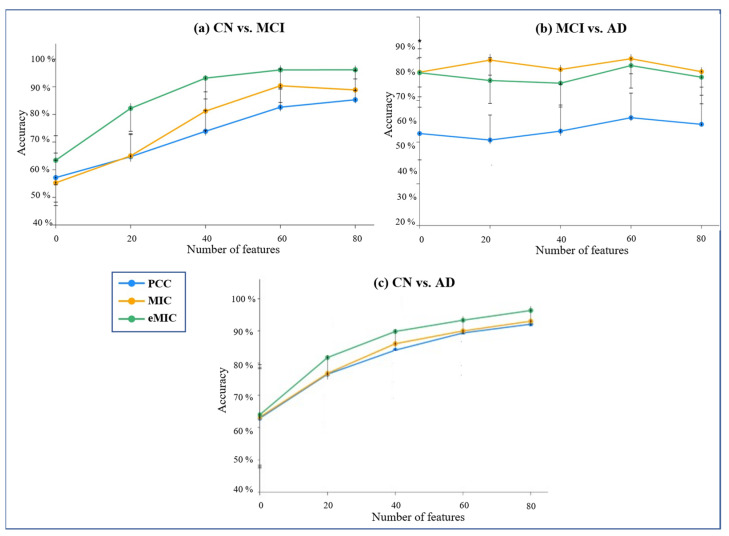
Classification accuracy graph using multilayer ELM. Horizontal axes represent the number of features. (**a**) CN and MCI. (**b**) MCI and AD. (**c**) CN and AD.

**Figure 6:**
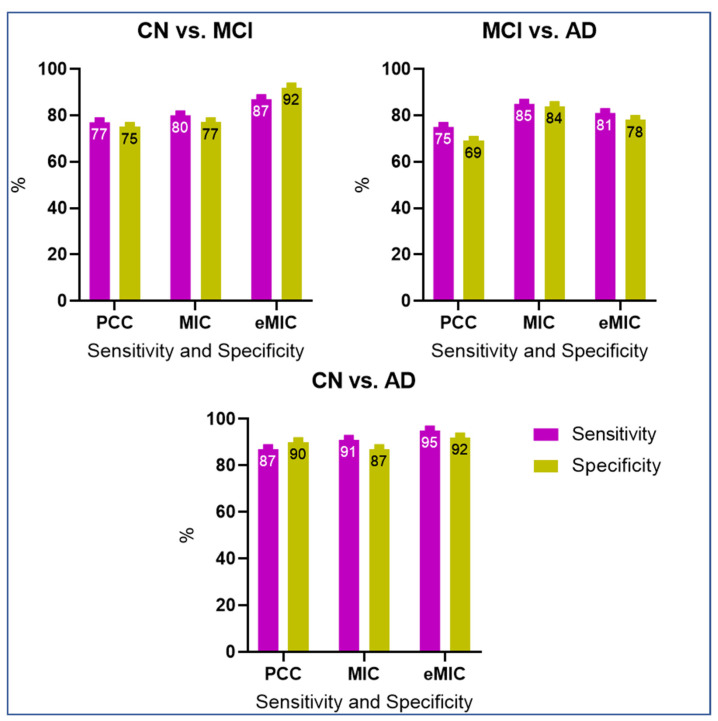
Sensitivity and specificity graph of AD classification. CN and MCI. MCI and AD. CN and AD.

Using different feature counts, we assessed the classification accuracy for CN versus MCI, MCI versus AD, and CN versus AD (Figure 5). To gain a more comprehensive evaluation of the results, we calculated the sensitivity and specificity of the classification outcomes (Figure 6).

The classification accuracy of CN versus MCI based on various feature counts is shown in Figure 4a. Features utilized by eMIC produced the highest classification accuracy. The features calculated using PCC, on the other hand, produced the lowest accuracy rate. The accuracy of the classification remained stable as the number of features increased.

Based on different numbers of features, the classification accuracy of MCI and AD is shown in the second figure, Figure 4b. However, the final classification accuracy for MIC- and eMIC-calculated features was not significantly different.

The classification accuracy of CN and AD is depicted in the third figure, Figure 4c. When eMIC-based features were utilized, classification accuracy was superior to that of PCC and MIC. The classification accuracy was the lowest when features derived from PCC were used. Based on eMIC features, the average classification accuracy for CN and AD was 95%, which was higher than MIC’s 90% classification accuracy. The classification made with PCC features had an average accuracy of 87%. PCC had the lowest average classification accuracy for MCI and AD classification (58%). Classification using MIC and eMIC features resulted in a relatively similar average accuracy rate of 81% and 78%, respectively. PCC had an average classification accuracy of 87% for CN and AD, which was lower than the 90% and 95% for eMIC and MIC features, respectively.

Based on features extracted from PCC, the sensitivity and specificity of classification for CN and MCI were 77 and 75%, respectively, as shown in Figure 5a. The scores for MIC and eMIC based classification were 80 and 77%, and 87 and 92%, respectively. As per Figure 5b, the classification of MCI and AD showed a sensitivity and specificity of 75 and 69.5%, respectively, for features acquired from PCC, 85 and 84% for features extracted from MIC, and 81 and 78% for features extracted from eMIC. As per Figure 5c, the sensitivity and specificity for classifying CN and AD using features from PCC were 87 and 90%, respectively, as compared to MIC’s 91 and 87% and eMIC’s 95 and 92%. Many studies have been carried out utilizing rs-fMRI to differentiate AD and MCI from CN. As stated previously, the proposed work achieves the best level of accuracy for AD classification (95% (CN vs. AD) using eMIC-based FC matrices.

Furthermore, extensive research has been conducted to investigate a range of neuroimaging techniques aimed at effectively distinguishing between AD and MCI. However, the direct comparison of our proposed method with state-of-the-art approaches is challenging due to inherent variations in datasets and classification methodologies employed across the existing literature. It is important to acknowledge that the inclusion of additional training and testing data in other studies introduces additional complexities in directly comparing our method. Notably, the current methodologies employ distinct features and feature selection strategies to explore various binary classifications, such as AD vs. CN, CN vs. MCI, or MCI vs. AD. These variations significantly impact the overall accuracy of performance. To provide insights into the differentiation of CN from AD and MCI, Table 2 presents accuracies reported in prior studies using binary classification, unique feature selections, and classifiers. The compilation aims to offer a comprehensive understanding of the classification performance across a variety of research studies.

In the research described in [43], the authors introduced a Bayesian Gaussian process logistic regression model for the classification of AD and MCI. The model incorporated features selected from FC measures combined with relevant phenotypic data, leveraging Kendall’s tau correlation coefficient. The study aimed to assess the effectiveness of the Gaussian System Logistic Regression (GPLR) template, a specific multivariate statistical machine learning software, in stratifying patients based on functional communication patterns throughout the brain during rest. The algorithm proposed in [44] integrates fMRI images with essential medical information, including age, gender, and genetic data. A stacked autoencoder architecture has been utilized to train a deep neural network using fMRI time-series data or correlation coefficient data. In the study carried out in [45], the authors computed integration and separation metrics from graph-based analyses. Feature selection was performed using Fisher scoring, and AD classification was carried out using SVM. In [46], functional brain states were estimated using the hidden Markov model (HMM) framework. The HMM pipeline encompassed the estimation of both the functional brain states and their dynamics. Each state was parameterized using a multivariate normal distribution, with a particular emphasis on analyzing the covariance matrix to interpret each state as a distinct connectivity pattern. The impact of the number of subjects on the accuracy of the tests is evident, with accuracy declining as the subject count increases. As mentioned earlier, our proposed work achieves the highest accuracy of 95% in classifying AD by employing a combination of eMIC-based correlation features and multi-layer ELM. In comparison to existing approaches, our study outperforms state-of-the-art methods in terms of the results obtained for MCI vs. CN and AD vs. CN classifications. However, it is important to note that direct comparisons of performance with other studies may not be fair or reliable due to variations in the datasets, preprocessing pipelines, features, and classifiers employed in each study.

### Limitations of This Work

The classification of medical images is a fundamental and significant issue in computer innovation that has undergone much research over the past few decades. Even though the reliability of various medical image classification methods has significantly increased, these methods may not offer correct AD because of their non-universality, vulnerability to illumination and spoofing effects, and insufficient accuracy via the poor data quality. Therefore, in many real-world applications, standard medical picture categorization may not be able to deliver the needed performance. In this study, we solely used the ADNI dataset to classify AD and MCI from cognitively normal controls. The dataset we used here is small for the entire experimentation. Additionally, this work solely uses new methods for feature extraction and AD classification, such as MIC, eMIC and MLELM, instead of alternative techniques.

## 5. Conclusions

Early identification of AD and MCI is critical for implementing preventive measures and slowing down the progression of the disease. This study aimed to classify AD, MCI, and CN individuals using both linear and non-linear FC features extracted from PCC, MIC, and eMIC analyses. Our findings reveal that the non-linear FC features exhibited superior performance compared to the linear features, demonstrating higher classification accuracy. This suggests the potential of non-linear FC measures as robust biomarkers for AD detection. Additionally, the non-linear features demonstrated more balanced behavior in the classification results, further highlighting their effectiveness in distinguishing between different cognitive states. By incorporating non-linear FC measures, this study contributes to enhancing the precision and accuracy of clinical AD classification, ultimately aiding in the early detection and management of AD and MCI.

Furthermore, the advancements in fMRI data analysis techniques showcased in this study pave the way for the development of advanced tools for medical diagnosis and treatment. These findings underscore the importance of utilizing non-linear FC measures in improving the understanding of AD pathology and facilitating more accurate diagnoses.

Future research should focus on investigating the longitudinal progression of AD and MCI, exploring the synergistic effects of combining multiple imaging modalities, and assessing the impact of non-linear FC measures on treatment response. These avenues of research will further enhance our understanding of AD and facilitate the development of more effective diagnostic and therapeutic approaches.

## Figures and Tables

**Table 1:** Dataset description.

Type	CN	MCI	AD	Total
Number of patients (M/F)	66/69	106/44	52/48	383
Average age (years)	77.25	75.21	75.1	75.8

**Table 2:** Comparison of CN differentiation from MCI and AD in recent works.

Authors	Methods	Subjects	Task	ACC (%)	SENS (%)	SPE (%)
Challis et al. [43]	Gaussian and regression models, covariance	20 CN/50 MCI/27 AD	AD vs. CN	80.12	70.97	90.24
			CN vs. MCI	75.15	100	50.52
Ju et al. [44]	Deep autoencoder	79 CN/ 91 MCI	CN vs. MCI	86.5		
Khazaee et al. [45]	Naïve Bayes and directed graph features	45 CN/34 AD	AD vs. CN	93.3		
Eavani et al. [46]	HMM, ICA and covariance matrix of whole brain network	31 CN/31 MCI	CN vs. MCI	62.9		
Proposed work	MIC/eMIC correlation-based FC, multi-layer ELM	135 CN/148 MCI/100 AD	AD vs. HC	95	95	92
			AD vs. MCI	81	85	84
			HC vs. MCI	94	87	92

## Data Availability

Not applicable.
